# Brucellosis Control in Malta and Serbia: A One Health Evaluation

**DOI:** 10.3389/fvets.2018.00147

**Published:** 2018-07-03

**Authors:** Sandra C. Buttigieg, Sara Savic, Daniel Cauchi, Elaine Lautier, Massimo Canali, Maurizio Aragrande

**Affiliations:** ^1^Department of Health Services Management, Faculty of Health Sciences, University of Malta, Msida, Malta; ^2^School of Social Policy, College of Social Sciences, University of Birmingham, Birmingham, United Kingdom; ^3^Scientific Veterinary Institute “Novi Sad”, Novi Sad, Serbia; ^4^Department for Health Regulation, Health Promotion and Disease Prevention, Ministry for Health, Valletta, Malta; ^5^Department of Agricultural and Food Sciences, University of Bologna, Bologna, Italy

**Keywords:** brucellosis, control, evaluation, Malta, one health, Serbia, zoonosis

## Abstract

Brucellosis, also known as “undulant fever” or “Malta fever”, is a zoonotic infection caused by microorganisms belonging to Brucella, a genus of gram-negative coccobacilli that behave as facultative intracellular pathogens of ruminants, swine and other animals. Brucellosis is a threat to public health, hence identifying the optimal way of preventing disease spread is important. Under certain circumstances, integrated, multidisciplinary “One Health” (OH) initiatives provide added value compared to unidisciplinary or conventional health initiatives. Conceptualizing and conducting evaluations of OH approaches may help facilitate decisions on resource allocation. This article historically describes and compares Malta's 1995–1997 with Serbia's 2004–2006 brucellosis control programmes and quantitatively assesses the extent to which they were compliant with a OH approach. For both case studies, we describe the OH initiative and the system within which it operates. Characteristic OH operations (i.e., thinking, planning, working) and supporting infrastructures (to allow sharing, learning and systemic organization) were evaluated. We scored the different aspects of these programmes, with values ranging from zero to one (1 = strong integration of OH). Malta demonstrated a higher OH index (0.54) and ratio (1.37) than Serbia (0.49 and 1.14 respectively). We conclude that context and timing are key to determining how, when and why a One Health approach should be applied. The adoption of a true OH approach that involved systemic organization, leadership clarity and transdisciplinary communication, collaboration, and co-ordination was essential to Malta's successful eradication of brucellosis after several failed attempts. In contrast, contextual factors in Serbia permitted the successful adoption of a primarily sectorial approach for short term control of brucellosis. However, while a fully-fledged transdisciplinary OH approach was not initially required, it is likely to be key to maintenance of brucellosis control in the medium and long term. Through these two case studies, we demonstrate that One Health initiatives should be applied at the right place, at the right time, with the right people and using the appropriate conditions/infrastructure. Lastly, OH evaluations should include economic assessments to identify optimal of resources in these situations, thereby justifying funding and political support required.

## Introduction

Brucellosis is one of the most common zoonotic infections worldwide, and remains a major public health concern (1–4). Known variously as “Mediterranean fever,” “undulant fever,” or “Malta fever,” the infection is caused by microorganisms belonging to *Brucella*, a genus of gram-negative coccobacilli that behave as facultative intracellular pathogens of ruminants, swine and other animals (4). Currently at least 8 species of *Brucella* are known, of which four, namely *Brucella melitensis, Brucella suis* and *Brucella abortus* and *Brucella canis* are known to have moderate to high human pathogencity. *Brucella melitensis—*the most frequent aetiological agent in sheep and goats (5)—is the main pathogen responsible for human brucellosis, followed by *Brucella abortus* and *Brucella suis*. The disease causes clinical morbidity in humans, as well as a considerable loss of productivity in animal husbandry in the developing world (5, 6). Animal infection is characterized by increased likelihood of abortion, impaired fertility and reduced milk production, with serious potential financial consequences for the individual livestock holder and communities (7). Humans are accidental hosts, who readily acquire brucellosis through consumption of unpasteurized dairy products; direct contact with infected animals, placentas or aborted fetuses; or inhalation of aerosols (4, 5, 7). The disease typically manifests in humans as an acute febrile illness which may progress to a chronically incapacitating illness with severe complications (5). Any organ and system of the human body may be implicated, yet it is often unrecognized and frequently goes unreported (4). Osteoarticular and reproductive disease are most common complications, whereas endocarditis remains the principal cause of mortality if the disease is not adequately treated by protracted, combined antibiotic administration (2, 4, 5). Relapses, at a rate of around 10%, may occur in the first year after infection as a result of inadequate treatment (5). Its duration and associated prolonged convalescence period means that brucellosis has important medical, as well as economic implications, such as infected persons' absenteeism from work. Brucellosis is considered to be an occupational hazard in shepherds, abattoir workers, veterinary surgeons, workers in the dairy industry, and microbiological laboratory personnel (4). Vaccination is the cornerstone of control programs in livestock; vaccines for cattle, sheep and goats have been developed, however a human vaccine for brucellosis does not yet exist (8).

Brucellosis is still endemic in many parts of the world, particularly where geographical and climatic conditions together provide the perfect medium leading to dissemination of the disease. Factors contributing to these conditions include poor grazing lands that do not permit the grazing of cattle (but are favorable for sheep and goats), and situations where farm animals are kept in close proximity to humans (9). The epidemiology of human brucellosis has changed drastically in recent decades as a result of political and socioeconomic factors, improved surveillance systems, animal-based control programs, and growing international tourism and migration (7, 10). While there are no reliable data on the global burden of brucellosis, a figure of 500,000 new cases per year is usually accepted as a global estimate (1). Although there has been significant progress in controlling the disease in many countries, areas where the infection persists in domestic animals remain. Consequently, transmission to humans is common, particularly in Mediterranean countries, north and east Africa, the Middle East, south and central Asia, and Central and South America (4). Few countries are officially free of the disease (1, 7).

The prevention, control and eradication of brucellosis typically require collaboration across a number of sectors (4). According to the One Health Initiative (www.onehealthinitiative.com), “One Health” is an umbrella term referring to the commonalities between people, animals, plants and the environment. It recommends integrative approaches to health by expanding interdisciplinary collaboration across these highly interlinked components (11–13). The participation of representatives from Malta and Serbia in the EU COST action TD 1404 “Network for Evaluation of One Health”—these being two countries where efforts to control or eradicate brucellosis have been mostly successful: in Malta during the last decade of the twentieth century after several failed attempts (9, 14), and in Serbia during the first decade of the twenty first century (15, 16)—led to this study. The objectives of this comparative study are 2-fold. First, we aim to provide a short historical account of the process and co-ordination of actions in both countries, and compare Malta's 1995–1997 with Serbia's 2004–2006 control and eradication programmes. It should be noted that contextual and temporal differences between the two countries led to the adoption of substantially different approaches to brucellosis control. Furthermore, in June 1999 the “United Nations Security Council resolution 1244” established the United Nations Interim Administration Mission in Kosovo (17). Subsequently, brucellosis data from Serbia did not include Kosovo. Any mention of Kosovo in this manuscript therefore refers solely to pre-1999 data, when Kosovo was administratively part of Serbia. Second, we will quantitatively evaluate “One Health-ness” of the programmes through the calculation of an index and ratio—developed by the Network for Evaluation of One Health—to assess the extent to which they were compliant with a One Health (OH) approach. For both case studies, we describe the OH programme or initiative (i.e., drivers, operations, supporting infrastructure and outcomes) and the system (i.e., dimensions, boundaries, aim, actors, and stakeholders) within which it operates (12). The major elements evaluated through the One Health framework are social, environmental, and economic in nature. Different characteristic OH operations (i.e., thinking, planning, working) and supporting infrastructures (to allow sharing, learning and systemic organization) are also examined (12, 13).

Successful control of brucellosis in Malta and Serbia was only achieved and maintained when the strategy to address the infectious disease in both countries demonstrated OH characteristics of leadership clarity and transdisciplinary communication, collaboration, and co-ordination. This evaluation is intended to inform scholars, practitioners, and communities involved in the surveillance, control and management of brucellosis about the salient features and potential usefulness of adopting the OH approach. Although, the evaluation is being conducted retrospectively, it should provide a useful roadmap prospectively.

### Historical account

The following is a historical commentary upon the two countries' brucellosis control strategies, approached primarily from a social and environmental perspective. In Malta, the eradication process was steered by the Ministry of Health's Public Health Department, whereas the Ministry of Agriculture's Directorate for Veterinary Medicine led the Serbian control initiative. This is primarily because Malta lacks an academic department for veterinary science, and control of infectious diseases is the legal responsibility of the Superintendent of Public Health. Brucellosis is a notifiable disease in both Malta and in Serbia, hence reporting the disease is mandatory if it is suspected or diagnosed in humans or animals.

#### Malta

Brucellosis (namely *Brucella melitensis*), had long been endemic in Malta, to the point where it was known as “Malta fever” (9). From an environmental perspective, Malta has poor grazing lands that are only favorable for the herding of small domesticated ruminants (sheep and goats]. The proximity of humans to these animals and regular consumption of unpasteurized goat milk was highly prevalent in Malta at the beginning of the twentieth century, resulting in a continuous potential source of infection for the general population. Despite seminal work on the pathogenesis of the disease carried out by Sir Themistocles Zammit—a Maltese doctor—which led to the identification of unpasteurized goat milk as the major source of infection in 1905 (18, 19), there was a lack of knowledge among the general population that goats were the primary reservoirs of infection. Furthermore, throughout the twentieth century there was persistent cultural resistance to the notion that unpasteurized goat's milk and related products—typically considered to be “healthy” and “wholesome”—were in any way related to the disease (9). Goat herders in particular were notoriously reluctant to comply with authorities, and a portion of the population persisted in consuming raw milk (9). This unwillingness among the population to change traditional behavior was the primary reason for multiple failed attempts at eradication during the twentieth century, in addition to a *Brucella melitensis* eradication programme launched in 1956 which was never properly implemented (9).

During the late 1980s, several Government departments—including the Public Health, Veterinary Services, Agriculture and Consumer Affairs departments—worked together in an attempt to secure the entire production chain of fresh cheeselets (small round cheeses made from milk, salt and rennet) and milk. Policy makers were highly engaged in the process and introduced effective legislation that required the registration of all herds in Malta. In 1987, the Veterinary Services Department (VSD) launched the “Test and Slaughter” scheme (20) across all milk-producing herds in Malta. Goats and sheep above 6 months of age were identified through ear tagging or freeze branding, thus facilitating a more effective, systematic 6-monthly screening process. Infected animals were slaughtered within 14 days. If more than 10% of animals in a herd were infected, the herd was depopulated and the farm disinfected (21, 22). The Director of Agriculture issued new regulations making it obligatory for farmers to notify any movement of animals from one farm to another and supported the tattooing, freeze-branding or ear-tagging of the animals (22). Between 1986 and 1996, prevalence of infection within herds fell from 23 to 1% (22). However, despite these initiatives, an outbreak of the disease occurred in 1995, when around 238 cases of human brucellosis were diagnosed (22). The 1995 outbreak revealed weaknesses in the system, demonstrating that more work needed to be done to achieve control of brucellosis.

Following the 1995 outbreak, an intersectoral outbreak committee was set up. The Ministry of Health and the Department of Public Health led the brucellosis eradication initiative of 1995–1997, which was characterized by interdisciplinary collaboration between the major stakeholders. These included public health inspectors, public health doctors, microbiologists, medical doctors, the police, and veterinary surgeons. A clear case definition for identification of brucellosis in humans was established. Any person presenting with one of the following symptoms: fever; weakness; headache; chills; arthralgia; localized suppurative infection or encephalopathy, who also had a *Brucella* antibody titer of > 1 in 320 dilution or a positive culture of *B. melitensis*, or who had a member of their household with a *Brucella* antibody titer > 1 in 320 dilution (with or without symptoms), was classified as a case. The Disease Surveillance branch of the Department of Public Health extensively sampled and tested cheeselets sold in shops, street vendors and supermarkets across the Maltese islands. The Department of Agriculture was subsequently notified regarding suspect herds, which were examined further and blood testing carried out (21). The main source appears to have been three farmers who kept so-called “phantom” herds concealed from routine VSD inspections. Further spread to other herds occurred when these owners fragmented their unregistered herds and sold them off cheaply in order to avoid depopulation (22).

The outbreak committee also communicated regularly with the general public and issued several press releases during this time. The Public Health Department also delivered a mass media educational campaign to foster awareness of the potential ill-effects of consuming unpasteurized cheeselets among the general public. Additionally, the Agriculture Department organized a series of talks delivered to farmers and herders that focused on hygiene and the importance of pasteurization. Detailed leaflets regarding the best method of manufacturing cheeselets were prepared. The national dairy company offered pasteurization services to the producers and created its own branded cheeselets, marketing them as “guaranteed safe” to reassure the public. Draft regulations were implemented to control the hygienic processing, transport and sale of cheeselets: new packaging and labeling practices required the introduction of a “lot” number, “best before” dates, the producer of the cheeselets and whether they were made from pasteurized or unpasteurized milk (9). The sale of fresh unlabeled cheeselets by weight was banned. Although the outbreak committee focused primarily on human infection, its efforts were supported by a highly active health inspectorate who confiscated 930 kg of cheeselets from 27 producers, 12 wholesalers and 384 retailers during this period, as well as VSD staff who destroyed 116 caprines, 68 bovines and 43 ovines after screening 3,416 herds in Malta and 1,449 herds in Gozo (9). Malta was declared free from locally acquired human brucellosis in 2005 (23), and from bovine brucellosis in 2016 (24).

#### Serbia

A lack of knowledge about the mechanisms of spread of *B. melitensis* and *B. abortus* among animals and in humans, and non-regulated import of infected animals from neighboring countries, characterized the Serbian scenario. In the former Yugoslavia, brucellosis was reported for the first time in the district of Istra in 1947, but was eradicated within a few years (15, 25). It reappeared in the 1960s, in Macedonia, probably through sheep imported from Israel (26). By the late 1970s, sheep-borne Brucellosis had appeared in most territories of Macedonia, in Kosovo and Metohija, as well as in south Serbia (27). While the epidemiological situation in the Republic of Serbia was relatively stable up to the 1980s, with around 40 human cases identified between 1951 and 1970 (27), this was not maintained over the following two decades. Incidence increased from 1985 and peaked in 1991 (25). During the 1990s, brucellosis spread to central and north Serbia as a consequence of armed conflicts and uncontrolled movement of infected sheep (15). The disease has also spread to south Serbia, in the region bordering with Kosovo and Metohija, in recent years (15).

A critical increase in cases of Brucellosis was observed in the territories of Kosovo and Metohija, with 241 cases being reported in 1991 (28). The disease also reappeared in areas that had previously been considered to be free of the disease. For example, brucellosis had not been diagnosed in either humans or animals in Vojvodina, a province in the northern part of Serbia, during a thirty-year period from 1971. However, a positive diagnosis was made in two farm workers in the South Banat district of Vojvodina in 1999 (15). Subsequently, foci of brucellosis continued to multiply and spread to neighboring counties, probably due to the uncontrolled movement of infected herds (e.g., illegal trade, nomadic livestock herding) compounded by poor implementation of countermeasures ordered by the Veterinary Service. Farm workers were exposed to infected animals, and consumers of milk products—such as cheese produced from unpasteurized sheep milk—were also infected. There was also some cross-species spread, as brucellosis was identified in other farm animals such as swine and dogs (28). By late 2004, new foci had been identified in five counties, and human brucellosis cases had been diagnosed in 12 settlements. Overall, 1,521 cases of human brucellosis were identified between 1980 and 2008 in Serbia (25).

The Ministry of Agriculture led the Serbian control programme of 1999–2005 through the Directorate of Veterinary Medicine, in collaboration with other actors including policy makers, veterinarians, medical doctors and police. In Serbia, the outbreak committee mostly focused on animals. An outbreak committee consisting of veterinary health specialists, veterinary inspectors, public health doctors, and microbiologists who established the case definition (i.e., any animal presenting with symptoms of fever, weakness, and/or abortions, with a positive antibody test of *B. abortus, B. melitensis*, or *B. suis*) was set up. During this period, a “test and slaughter” programme similar in scope to that described for Malta was established. Overall, the veterinary services destroyed 1,497 animals (cattle, pig, sheep and goats) in the northern part of Serbia after screening 1,485,702 animals. No data is available for the southern part of Serbia. The number of infected humans and animals significantly decreased in northern Serbia (Vojvodina province) after 2006, and in southern Serbia after 2009, and overall Brucellosis incidence now shows a declining trend (16). In Serbia, brucellosis may still occur in animals if these are illegally imported in the country (7). Controlling the trade in animals is likely to be a key method of controlling and preventing the spread of brucellosis (25). However, there are reports that wild boars and rabbits are reservoirs of *B.suis*, whereas dogs are reservoirs of *B. canis* in Serbia (29).

## Methods

We applied methods developed by the EU COST action TD 1404 “Network for Evaluation of One Health” (NEOH, http://neoh.onehealthglobal.net) (12). The NEOH evaluation framework is a mixed method approach that covers the definition of the initiative and its context, the theory of change (TOC), the process evaluation of operational and supporting infrastructures (“the One Healthness”), and an assessment of the association(s) between the process evaluation and the outcomes produced (12). This comparative case study *retrospectively* identifies drivers, outcomes, operations and infrastructure of the One Health approach to Brucellosis eradication and control (as applied in Malta and Serbia respectively) in an integrated manner, namely through the holistic assessment of these aspects. This analysis includes a historical account intended to offer insight into the geopolitical context of Brucellosis outbreaks and to identify and delimit the systems within which the OH initiatives were developed to differing extents. The TOC (30) underlies this process. To aid our analysis, we developed a visual approach for system identification and delimitation (see Figures 3, 4, below) and the further identification of costs related to Brucellosis in humans and animals (Figures 6, 7).

### Theory of change

**Figure 1:**
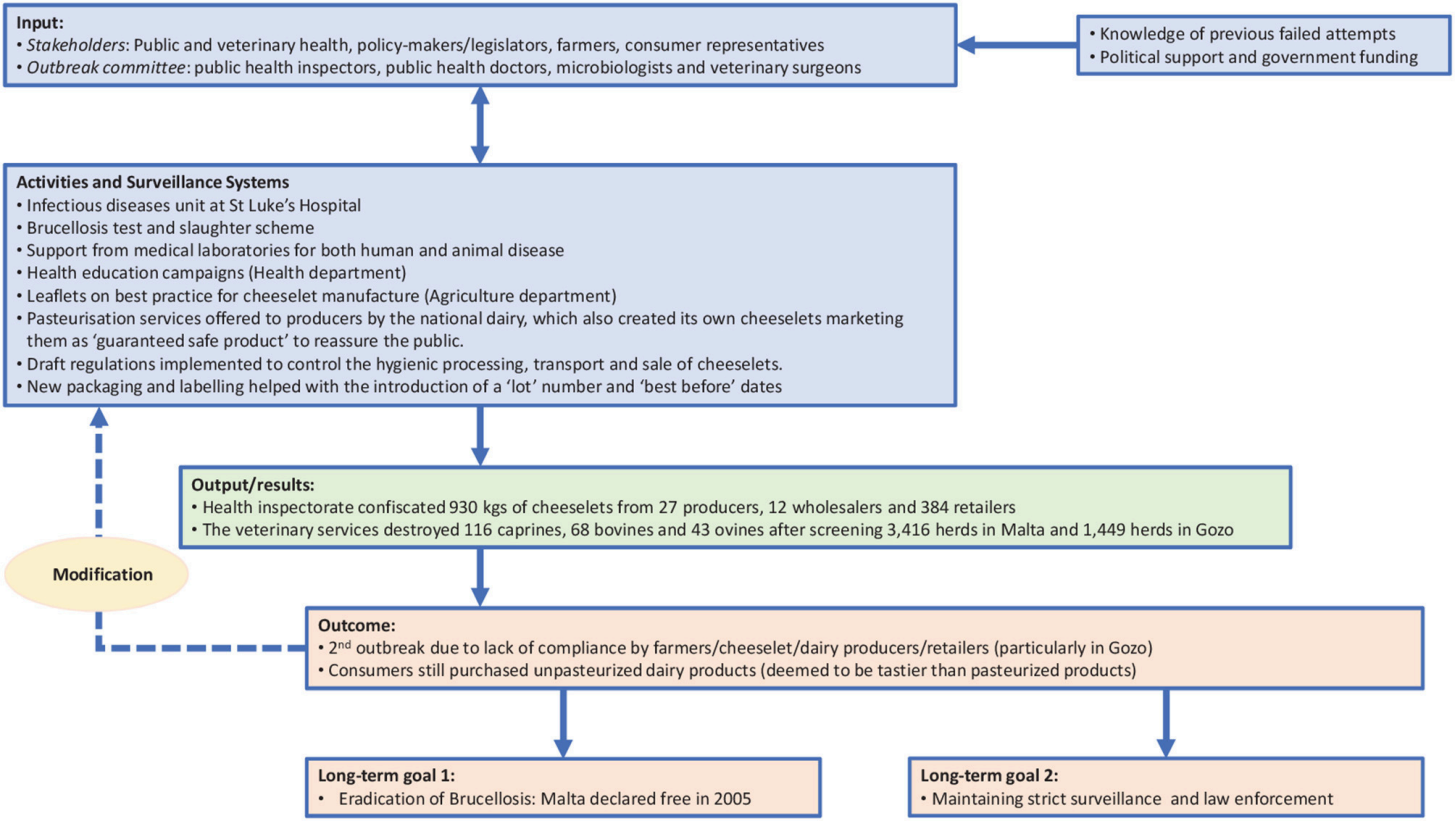
Pathway of Change representing TOC in brucellosis eradication in Malta.

**Figure 2:**
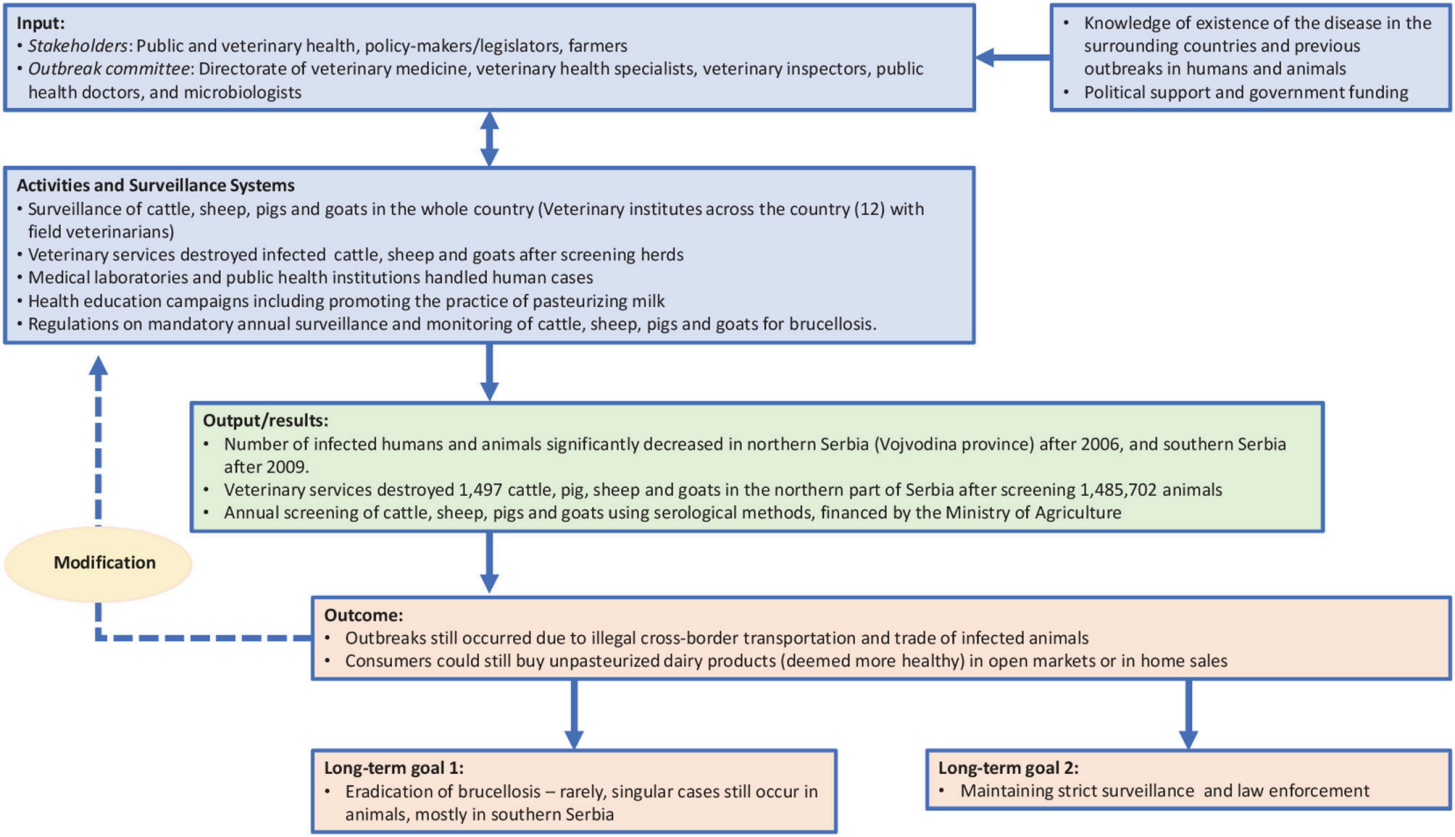
Pathway of Change representing TOC in brucellosis control in Serbia.

Within a OH approach, the TOC defines the objectives of the initiative, as well as the changes required to achieve these goals (13). Therefore, in line with NEOH guidelines, the TOC for brucellosis eradication and control provides a conceptual framework that enables retrospective analysis of the control and eradication committees' actions in both countries and definition of the short-, medium-, and long-term objectives that ultimately led to successful control (in Serbia) and eradication (in Malta). Figures 1, 2 illustrate the pathway of change (representing the TOC) applied to brucellosis eradication in Malta between 1995 and1997 (Figure 1), and brucellosis control in Serbia between 2004 and 2006 (Figure 2). For inputs, activities and surveillance, Serbia mainly relied on the veterinary services and lessons learnt from other countries. Although the 2004–2006 brucellosis outbreak in Serbia was ultimately controlled, the risk of infected animals being illegally brought into the country remains high, hence it is difficult to declare the country entirely free of brucellosis.

### Search strategy

A non-systematic literature search around brucellosis was conducted between January 2016 and April 2016 using Google Scholar, PubMed and EMBASE to identify scientific articles and gray literature that would inform the background of this study. The following search terms and key words were used: Brucell^*^ AND Malta OR Serbia. Furthermore, the bibliography of articles emerging from the search were reviewed to identify additional potentially relevant literature.

### Data collection

Using a case study approach, the authors obtained information primarily through 15 documented interviews carried out in both countries. In Malta, these interviews were recorded and transcribed by John Rizzo Naudi prior to 2005 (9) and involved key stakeholders across several disciplines, including main actors in animal health, human/public health and food safety/consumer health. A mix of telephone and face to face interviews with key stakeholders in Serbia were conducted by SS between 2015 and 2017. In both Malta and Serbia, a purposive sampling approach was adopted. This is a non-probability sampling technique, used when there are limited primary data sources available, that was judged by the researchers to be the most appropriate in order to identify relevant stakeholders in the brucellosis control programmes. All participants consented to be interviewed. With regard to animal health in particular, officials from the Veterinary Services Department (Ministry of Agriculture), veterinary service practitioners, and farm/animal owners in Malta were interviewed; whereas the Directorate of Veterinary Medicine (Ministry of Agriculture) as well as veterinary service practitioners and farm/animal owners were the interviewees in the case of Serbia. In view of potential biases arising from interviewees by virtue of the discipline they represent, we triangulated the data and information arising from the interviews using document analysis of legal documents, archival material from public health and veterinary sources, and other published material (9, 15). Document analysis is a component of qualitative research that involves in-depth assessment and interpretation of documents so as to substantiate the accounts provided by the interviewees (31).

We therefore retrospectively identified and discussed the various steps and systemic changes that needed to take place for brucellosis eradication or control to be achieved. Assessment of the following aspects: Thinking, Planning, Working, Sharing, Learning and Systemic organization—was conducted. The dimensions within each aspect—where each “dimension” refers to an entity that can be captured by the same metric or concept, such as geographical space or time—were scored in increments of 0.2 (where 0 = not considered; 1 = essential) by SB for Malta and SS for Serbia. Information on the scales for the different dimensions can be found in the appendices. Scoring of the NEOH evaluation tool (13) was then carried out by a focus group involving six professionals involved in public health and veterinary science in both countries. SB and SS were participants in this focus group. Ultimately, comparable OH-indices and ratios for the initiatives in Malta and Serbia were derived.

Lastly, a conceptual essay for economic evaluation that assesses the flow of cost and benefits is provided. This offers a basis for further evaluation aiming at assessing the advantages of the OH approach in comparison with traditional approaches toward disease control and eradication.

## Results

The Public Health Department (Ministry of Health) was the main actor for Malta, whereas the Directorate of Veterinary Medicine was the main actor for Serbia. Public health services in both countries included reference laboratories for human diagnostics; physicians, and hospitals. The Public Health Department assumed responsibility for these services in Malta, whereas Local Health Authorities were responsible for public health services in Serbia. Food safety/consumer health was only relevant to the Maltese scenario and involved the Superintendence of Public Health and Department of Consumer Affairs. Other major stakeholders included health education/promotion and policy makers from the Health, Agriculture, Justice and Internal Affairs Ministries in the case of Malta; and policy makers from the Ministry of Agriculture and Ministry of Internal Affairs in the case of Serbia.

### System dimensions and boundaries

**Figure 3:**
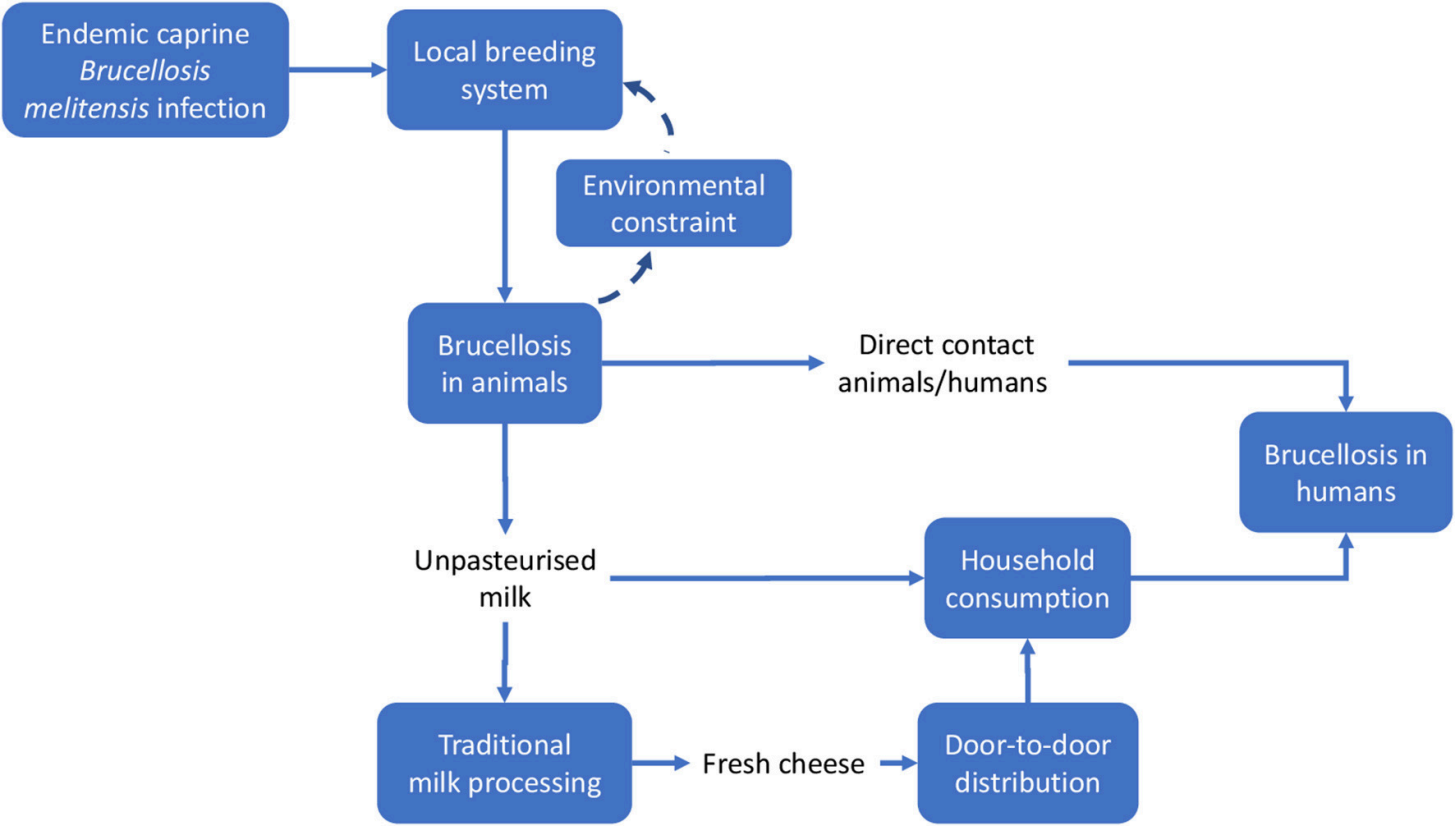
System identification in Malta. Solid lines indicate direct relationships or flows between elements; dashed lines stress potential reinforcement effects (feedback) of local brucellosis reservoir due to the use of pastureland.

**Figure 4:**
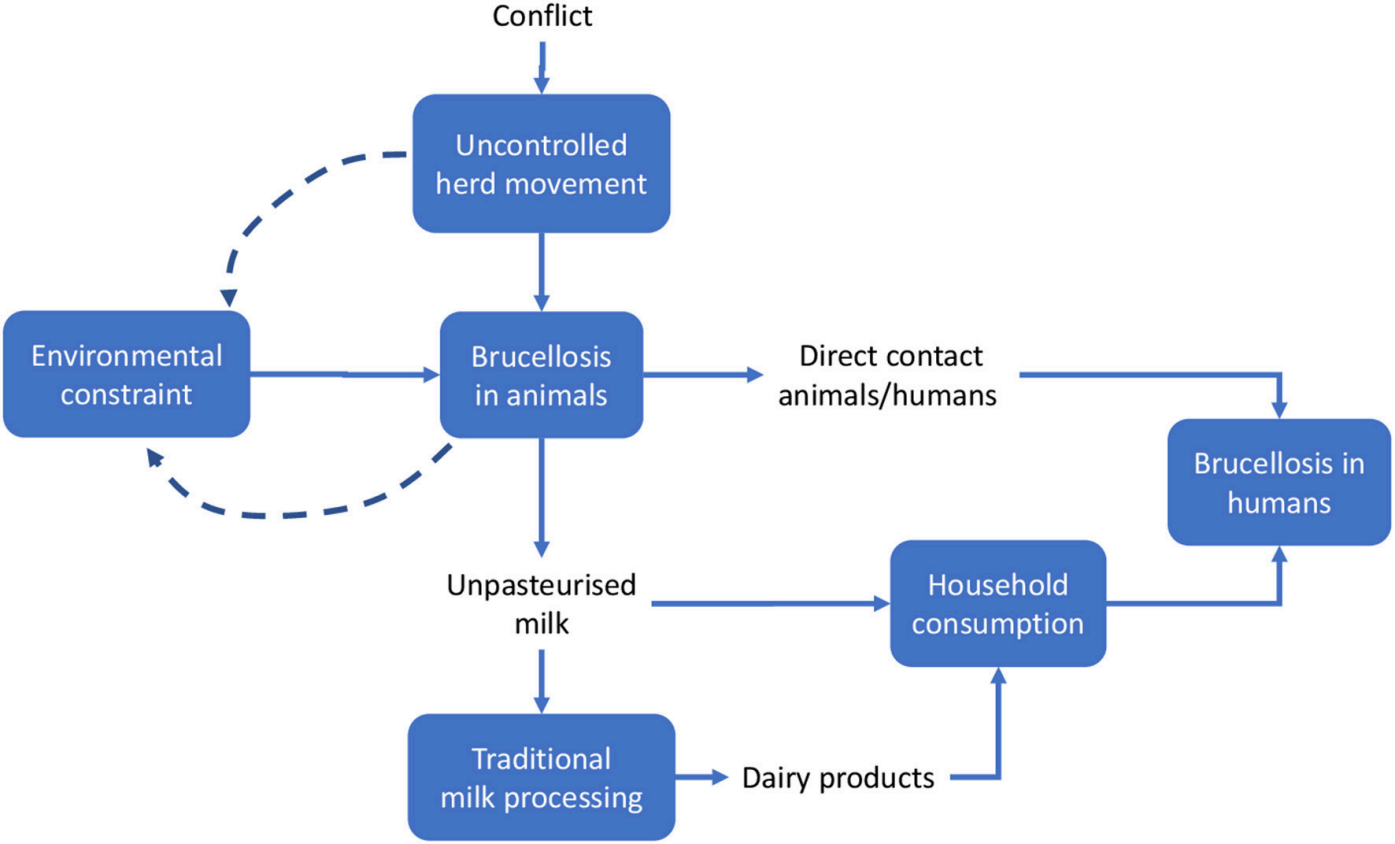
System identification in Serbia. Solid lines indicate direct relationships or flows between elements; dashed lines stress potential reinforcement effects (feedback) of local brucellosis reservoir due to the use of pastureland.

Meadows and Wright define a system as a “*set of elements or parts that is coherently organized and interconnected in a pattern or structure that produces a characteristic set of behaviors, often classified as its ‘function’ or ‘purpose.”’* (32). The application of this definition to the two country cases being analyzed led to the identification of the main elements that determined the emergence of the disease and its perpetuation. We outlined the system and the system boundaries in the two cases using a comparative approach, stressing similarities and differences. Figures 3, 4 schematically visualize the basic epidemiological models in Malta and Serbia respectively. We expanded the basic scheme of an epidemiological model to outline links and feedback loops connecting the emergence of brucellosis to the overall systemic contexts, identifying in particular both environmental and social factors. In the case of Malta (Figure 3) the presence of Br. in herd animals is a consequence of its endemic presence and the specific characteristic of the breeding system, based on the use of environmental resources (feeding on pastureland) where Br. is spread, reinforcing the emergence of the disease (blue dashed arrows). Environmental, breeding and distribution sub-systems transmit the disease to human beings *via* direct contact and food (i.e., the use of non-pasteurized milk in processing). Social practices and behaviors, as well as the general state of knowledge about mechanisms of disease spread, are of relevance to the wider socio-cultural sub-system which determines the insurgence and the persistence of the disease in the society (i.e., interaction between humans and animals inside and outside breeding places; traditional food distribution and consumption habits; and the misleading representation of product authenticity).

Other relevant elements of the system could not be shown in Figure 3, namely:

–The institutional framework, i.e., the institutions charged with solving the *Br*. problem, their organization and strategies (i.e., policy measures). The institutional framework can be considered to be a sub-system permeating and affecting the functioning of the basic system outlined in Figures 3, 4, thus enlarging its boundaries.
–The evolution of the institutional framework over time. Together with geographic space, time is a relevant dimension of the system, particularly because of the sequence of repeated attempts to control *Br*., the accumulation of scientific knowledge and of what was effective (i.e., through the failures of prior intervention measures) and the increase in social awareness, finally leading to the implementation of the OH-like model of intervention.

In the case of Serbia (Figure 4), the same approach was adopted, but some differential elements contributed to the identification of the system.

Based on the system identification process outlined above, we developed a comparative approach between the two cases. General similarities between the two countries include the basic contextual units of the system, such as the general epidemiological features of the disease and—assuming that the geopolitical territory of Malta and Serbia respectively represents the spatial limits of each system—its dissemination to human beings via direct contact and through the food supply chain (i.e., milk or milk products such as consumption of unpasteurized fresh cheese). Additional similar elements include:

- Local breeding systems reliant on the use of pastureland by different herds moving across the countryside (which promotes the dissemination of the disease within farms or family production units), characterized by prolonged close contact between animals and humans;
- The health system, in particular the lack of effective counter-measures and governance to address brucellosis;
- Limited knowledge regarding the risks related to brucellosis among the general population, which in turn determines local practices and behaviors in production, processing and consumption. In the case of Malta, this explained the social mis-representation of dairy product safety and led to the persistence of traditional processing and consumption practices, despite health measures implemented several times over a number of decades.

On the other hand, some features uniquely characteristic to each country may explain, at least in part, differences in the timeline and key characteristics of brucellosis development in the Maltese and Serbian systems. In particular, the unique geographical characteristics of the two countries resulted in different patterns of disease emergence and resilience to eradication and control, particularly in combination with:

- The differences in the political context (e.g., in Serbia, movement of people and herds during the conflict made it difficult or impossible to control the importation of infected animals from neighboring countries)
- The differences at institutional or organizational levels (i.e., animal health research capability; human health systems and public health systems)
- The greater relevance of traditional consumption habits in Malta, in comparison to Serbia, which resulted in a greater emphasis on the food supply chain for OH initiatives in that country
- Last but not least, Malta had a pioneering role in the discovery of brucellosis epidemiology. This probably contributed to the differences in timing and method of intervention strategies between the two countries.

A further step in system identification concerns the institutional and governance aspects of the health measures adopted in 1995–1997 and 2004–2006 in Malta and Serbia respectively. While Serbia's strong Veterinary Services played a leading role in the control of brucellosis, Malta's veterinary services were not developed to an equivalent extent and hence could not lead the control and eradication programme. Instead, Malta relied on a historically powerful public health sector, which was in a position to take on a leadership role in the most recent outbreak. These have been amply described in this paper.

Table 1 below synthetically compares the relevant systemic elements of the case studies.

**Table 1:** Synopsis of case studies comparison.

**Relevant elements/sub-systems of the system**	**Malta**	**Serbia**
Br. origin	Endemic, probably imported	Uncontrolled herd flows due to regional conflict
Breeding system	Family breeding, pre-industrial Transhumance	Family breeding, pre-industrial Transhumance
Environmental system	Use of common pastureland	Use of common pastureland
Processing system	Use of unpasteurized milk to produce traditional cheeselets	Use of unpasteurized milk in dairy products
Transmission mechanism	Direct contact	Direct contact
Social and cultural system	Traditional consumption habits Mis-representation of product authenticity Lack of scientific knowledge Lack of social awareness	Traditional consumption habits Lack of social awareness
Institutional system	Department of Public and Environmental Health as leader, in close collaboration with Departments of Agriculture and Veterinary Services, consumer Affairs, Justice and Police	Directorate for Veterinary Services as leader and prime mover, collaborating with Public Health, and Police
Policy and measures	*Laws of Malta*: Measures for the eradication of Brucellosis, Tuberculosis and Leucosis S.L. 437.86. Law transposed into policies across Government Departments for continued control and surveillance. The scope of these rules is to implement the rules contained in the European Union Council Directive 77/391/EEC concerning the introduction of Community measures for the eradication	Record keeping on brucellosis cases exists since 1984, when the Law on Infectious Diseases was passed. European Union (EU) has implemented various laws and restrictions regarding import and export of cattle and pig (EC 64/432), sheep and goats (EC 91/68), as well as regulations regarding products of animal origin, animal identification, and tagging

### Drivers and rationale

The rationale of the eradication and control processes in both countries was to address the infectious disease, with efforts primarily focused on systemic organization, leadership clarity and transdisciplinary communication, collaboration, and co-ordination. The following drivers spurred the intensity of efforts to control brucellosis in the two countries:

- *Economical*: high health care cost of treating human brucellosis; costs of surveillance; costs of government subsidies to farmers whose animals are eliminated because of the disease
- *Emotional/Psychological*: suffering of patients (humans, animals) affected by brucellosis; suffering of family and friends, particularly in fatal cases. Human cases of brucellosis were more prominent in Malta than in Serbia, where the disease seemed to have caused substantial emotional and psychological distress
- *Geographical*: Malta's island status contributed to the recognition of disease vectors and also helped to contain and maintain eradication of the disease. This driver was more of a challenge in Serbia, since the importation of infected animals was facilitated by porous land borders. Hence geographical location is a crucial consideration—Serbia depended on the actions of neighboring countries to manage its Brucellosis control process, whereas this was not the case for Malta
- *Social*: Malta's sister island—Gozo seemed to be less receptive to public health warnings regarding brucellosis, as manifested by the lingering belief that aseptic (clean) farming and retail environments were sufficient to ensure food safety of milk/products. The social driver in Serbia was primarily related to the country's post-war relations with neighboring countries and the lack of communication and trust between people from different (Former Yugoslavia) regions.

### Evaluation of “one health-ness”

**Figure 5:**
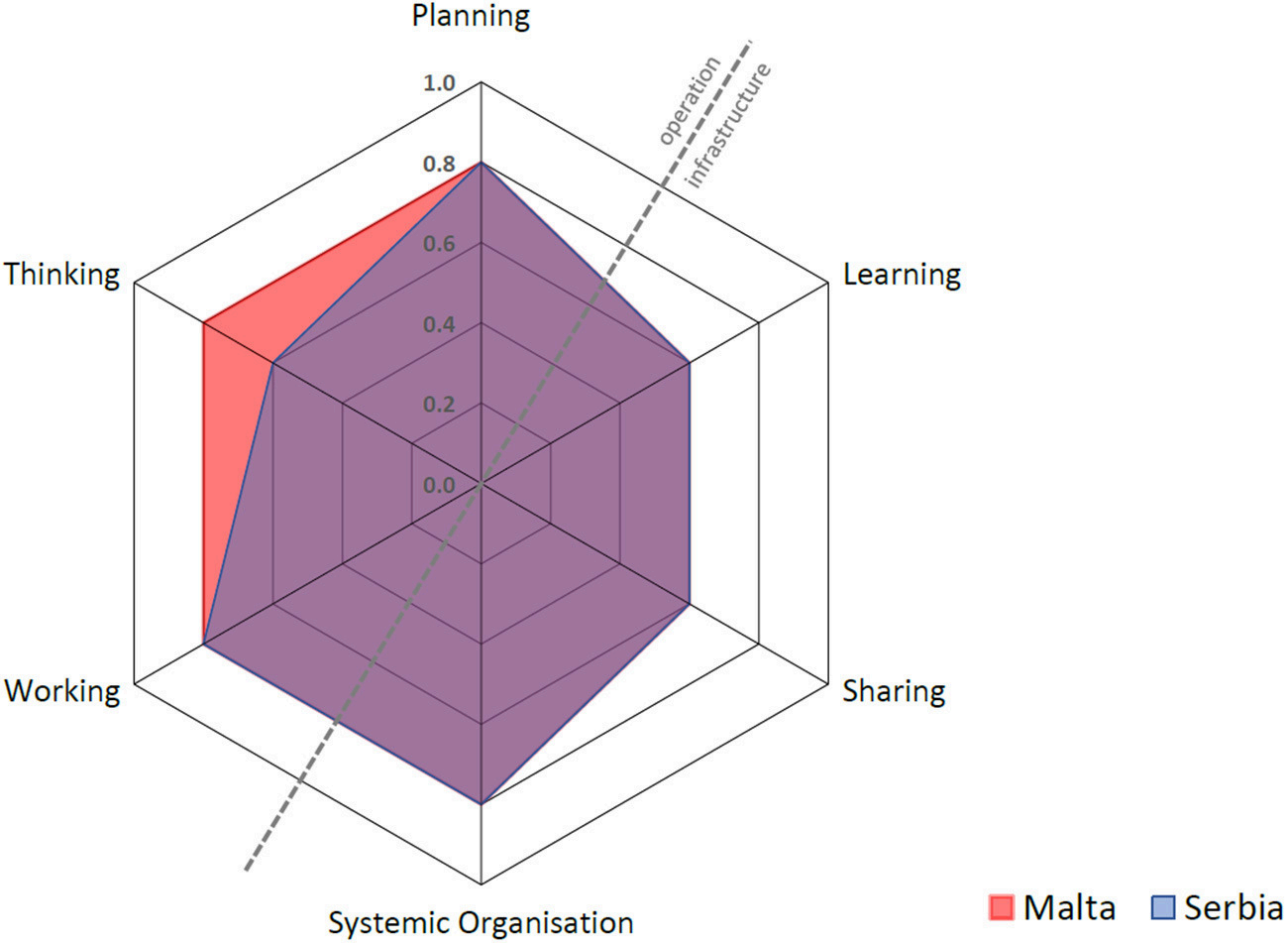
Malta and Serbia's One Health Index for the process of brucellosis control.

This section of the results deals with the quantitative evaluation of the “One-Health Index.” Each of the six assessments outlined below is represented by a spoke in the spider diagrams for Malta and Serbia (Figure 5) where thinking, planning and working (operational aspects) on the top left of the diagonal contrast with learning, sharing and systemic organization (infrastructural aspects) on the bottom right. The hexagonal surface represents the degree of integration, calculated as the One Health Index (OHI), whereas its symmetry or otherwise represents the balance between the operation and the supporting means of the OH initiative. This symmetry is numerically represented as the One Health Ratio (OHR) (13). Each assessment and its component dimensions are outlined in further detail below and in the appendices. Figure 5 shows that Malta and Serbia had identical scores for all assessments except for thinking, where Serbia scored lower. The details of the workings pertaining to Malta and Serbia can be found in the Supplementary Material.

#### Thinking

Thinking refers to the way actors and stakeholders think within and about the system and the One Health initiative. This includes an assessment of how the dimensions and scales under consideration (e.g., local, regional or global scales within geographical space; an understanding of the timeframe of the initiative; life; network or organization; economy; legislation; governance; and value constructs such as interest groups) may support or limit the outcomes and impacts of the initiative. The overall scores are 0.80 for Malta and 0.60 for Serbia. A major strength for both countries was the integrated health approach adopted during the eradication process. The lower overall score for Serbia is attributed to the lower sub-scores in the different dimensions' coverage and balance, and to sustainability and socio-ecological considerations. The focus in Serbia was timely control of the disease in animals before it spread to humans, hence fewer dimensions were covered and less importance was given to sustainability once there was successful control and effective law enforcement by the Veterinary Service. Although Malta's score for thinking is higher, this was mainly due to Malta's previous failed attempts at eradicating the disease, and reflects the fact that more stakeholders needed to be involved in order to finally achieve success.

#### Planning

One Health planning requires that aims, problem formulation, responsibilities, resource allocation and financing of the initiative are systematically organized. It also requires clarity in establishing roles, tasks, responsibilities, and competencies of participants (13). In this case study, this included consideration of whether stakeholder engagement during the process of Brucellosis control and eradication was planned, and whether mechanisms existed to feedback stakeholders' knowledge into the governance of the initiative. Such questions and other elements underpin the OH approach and contribute directly to OH outcomes, therefore planning may influence other assessments of the OH initiative under consideration (such as working, sharing, learning and systemic organization). The overall score for both Malta and Serbia was 0.80. In the case of Malta, the main focus of planning during the eradication process was the control of human disease and protection of consumers (i.e., from ingesting infected dairy products) while attempting to eradicate brucellosis in animals. In contrast, the major focus for Serbia included identification and registration of animal herds, rigorous blood sampling of animals and strict annual surveillance, led by the veterinary services.

#### Working

This assessment explores the extent to which engagement in the OH initiative was interdisciplinary and participatory (i.e., transdisciplinary) (13). Transdisciplinarity relies on appropriate leadership and management (i.e., system organization) to promote the establishment of non-hierarchical relationships, strategic dialogue, and shared decision-making between team members coming from different disciplines. The overall scores are 0.80 for both Malta and Serbia. Malta scored higher on collaboration between all the major stakeholders involved in policy, human health, animal health, retailing and consumer protection. Serbia scored higher for flexibility and adaptation, reflecting the successful leadership of the veterinary services.

#### Sharing

Sharing refers to the information and data-sharing infrastructures in One Health initiatives (13). Elements that were considered in this assessment include whether appropriate internal or external mechanisms were used for sharing information; whether resources were allocated to facilitate and ensure sharing of data; and what mechanisms in place for safeguarding access to data. The overall scores are 0.60 for both Malta and Serbia. Malta's scoring showed some resistance in sharing data and information, which partly explains prior failed attempts and the difficulties with law enforcement, particularly in Gozo.

#### Learning

The learning infrastructure within the One Health initiative comprises the learning style (i.e., whether basic, adaptive or generative) and setting (i.e., at the individual, team and organizational level). It also encompasses the type of environment: namely the stakeholders involved (“direct” environment), and the cultural, economic, and political situation surrounding the OH initiative (“general” environment). Our assessment considered whether these learning styles and environments supported a OH approach. The overall scores are: 0.60 for Malta and 0.60 for Serbia. Malta showed slightly greater emphasis on adaptive and generative individual, team and organizational learning, as a result of the crisis that ensued following the emergence of Brucellosis in humans.

#### Systemic organization

This assessment probes whether implementation of the OH initiative was facilitated by change-oriented leadership and effective teamwork, and therefore is closely related to and influenced by OH Planning. The overall scores are 0.80 for both Malta and Serbia. Despite differences in the methods leading to control and eradication, both countries manifested a rather strong sense of systemic organization as reflected by social and leadership structures and skills, team structures, competence and focus on innovation.

Table 2 shows that the overall Index and Ratio are slightly higher for Malta. This is attributed to the higher score of “thinking,” as well as to the greater degree of transdiciplinarity.

**Table 2:** “One-Healthness” of the systems in Malta and Serbia.

	**Malta**	**Serbia**
One health index	0.54	0.49
One health ratio	1.37	1.14

### Measured or estimated outcomes of the initiative or programme

#### Malta

Following the 1995 outbreak in Malta and subsequent efforts to eradicate the disease, there have been no cases of brucellosis in humans recorded since 1997. The control of the process, including monitoring and pasteurization of milk and cheese production and the enforcement of labeling and packaging is now co-ordinated by four collaborating departments: The Veterinary Services Department, the Public Health Directorate, the Agricultural Department and the Department for Consumer Affairs. Sharing and linking of information in inter/trans-disciplinary groups was well established in the 1995–1997 outbreak, which ultimately led to successful eradication. The information was successfully shared by representatives of disciplines on the outbreak committee and also to the non-scientific communities through information packages released by Ministries of Health and Agriculture.

Seminars and education activities are organized in order to increase the knowledge on the disease and involve stakeholders in surveillance activities, e.g., seminars and courses targeting official veterinarians and practitioners, medical doctors in hospitals and family doctors, and educational outreach campaigns targeting the general public.

#### Serbia

Sharing and linking of information between the Ministry of Health and Ministry of Agriculture was not carried out officially. While annual reports on zoonotic disease cases in humans (including brucellosis) are publicly available online, annual reports on animal screening from the Directorate of Veterinary Medicine are not publicly available. The yearly prevalence of brucellosis in animals can be found on the web site of the World Organization for Animal Health. Therefore, in contrast to Malta's isolation as an island state, which facilitated control of Brucellosis, Serbia remains susceptible to importation of the disease unless strict, vigilant border control is continuously maintained.

### Conceptual framework for economic evaluation

**Figure 6:**
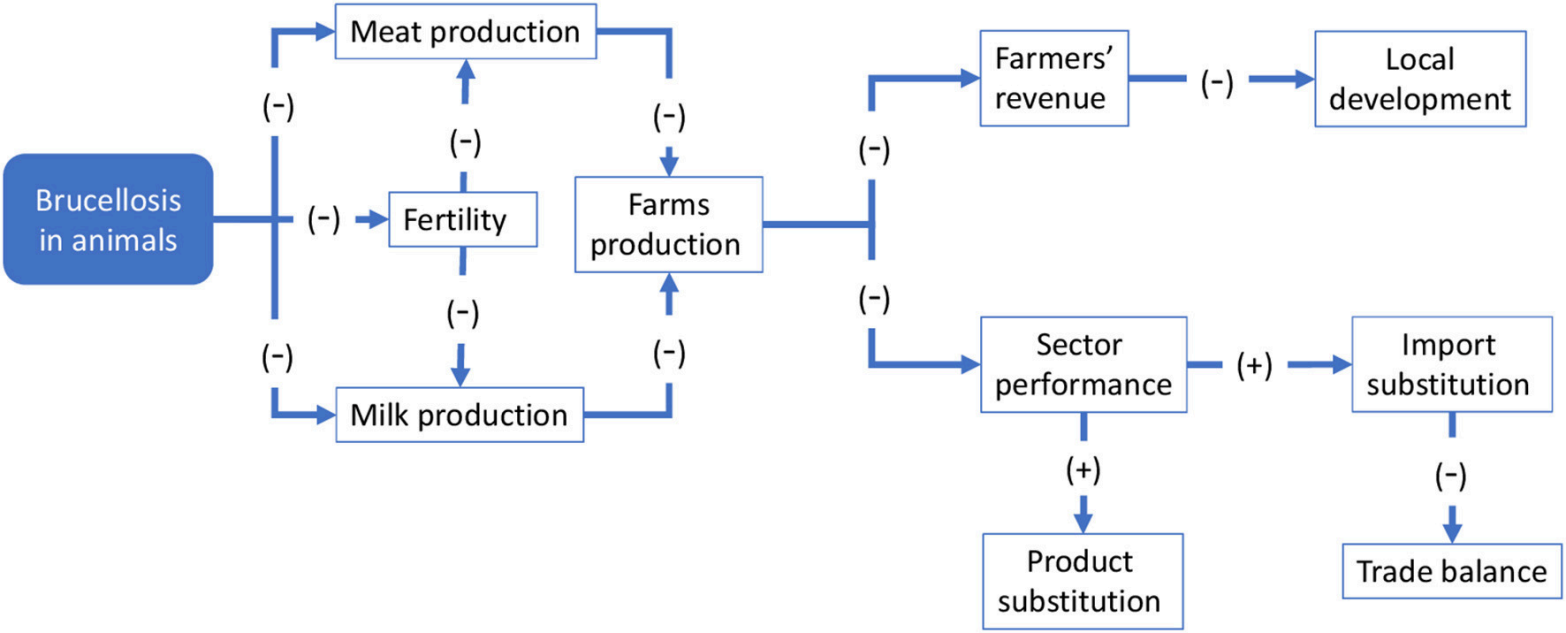
Economic consequences of brucellosis at farm and sector level.

**Figure 7:**
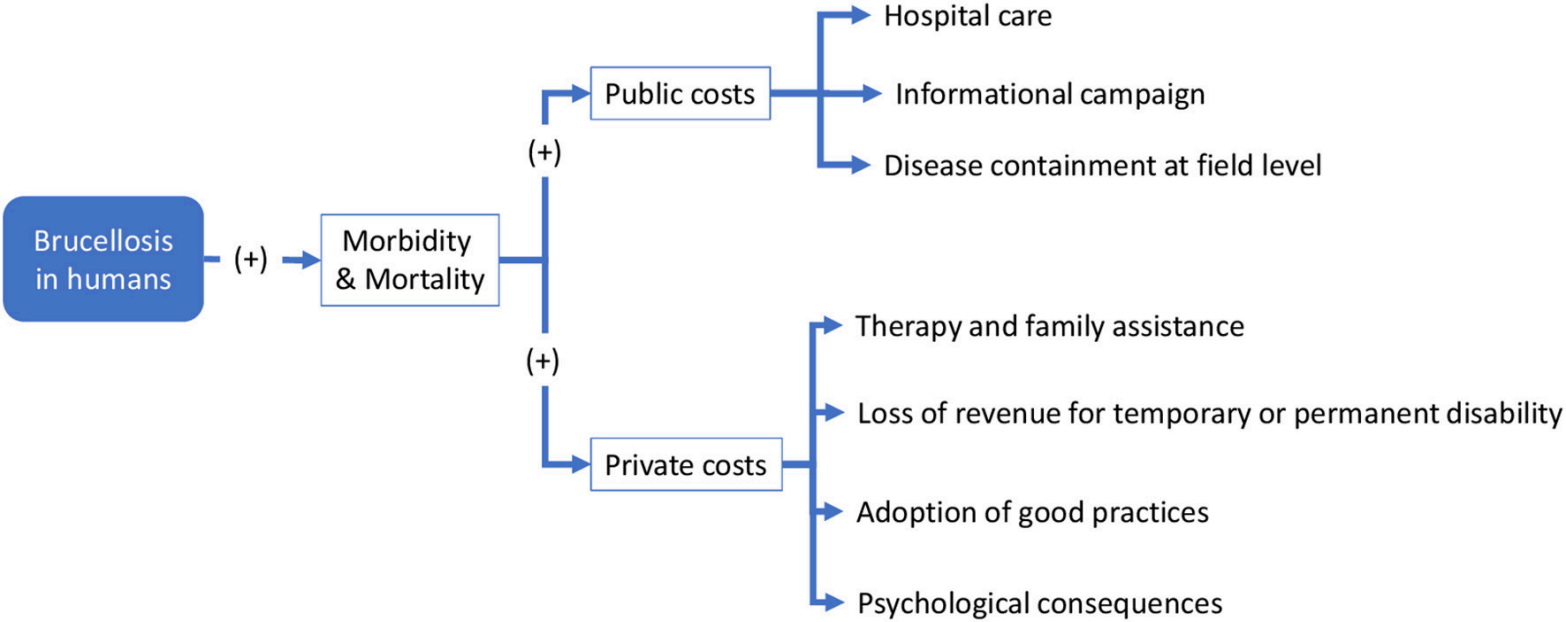
Economic consequences of Brucellosis across society.

This section focuses on the economic outcomes of brucellosis. It is likely that political support is more forthcoming when the adoption of an approach like One Health translates into economic gains. Although this study involves a conceptual, rather than an empirical, economic evaluation, potential economic impact of brucellosis may be identified through the schematic models in Figures 6, 7. For the sake of simplicity, we will start by making reference to the common traits of the system outlined for the two cases, assuming a static view concerning the pre-OH initiative scenario. Economic impacts are described starting from the boxes “Brucellosis in animals” and “Brucellosis in humans” of Figures 3, 4 above respectively—these represent the relevant outcomes of the epidemiologic models in Malta and Serbia, and the critical points of economic impact for the breeding system and the social system (i.e., households), according to the dissemination mechanism. White boxes in Figures 6, 7 represent the series of sequential effects stemming from Br. in animals and humans (indicated by the blue arrows between boxes). Signs in brackets show the direction of the effect (positive or negative) on the subsequent effect (see detailed explanation below). In particular, brucellosis in animals is responsible for the flow of effects along the food supply chain, starting from primary production and finally affecting consumers through processing and distribution). Brucellosis in humans concerns the effects of human infection across society (stemming from the consumption of unpasteurized milk and cheese and from direct contact between animals and humans).

Figure 6 outlines the potential economic effect of brucellosis at farm and sectoral level (where the sector is composed of the multiplicity of farms that breed sheep and goats). Brucellosis in animals leads lowered milk and meat production [as shown by (−) signs in brackets] due to premature births in infected animals. Fertility is also impaired, resulting in a lower natality rate that in turn also negative impacts milk and meat production. These effects result in a global reduction of farm production, which negatively affects sales and farmers' revenue. Depending on the relevance of the breeding to the local economy, this may have broader economic implications. At sectoral level, it could result in a global reduction of output, leading to a decline in competitivity of the local sector in comparison with other production areas. Consumers' welfare may be reduced, including through reduced product availability or diversity (not mentioned in Figure 6), but higher prices may induce substitution imports of similar products from elsewhere and/or determine product substitution [i.e., consumers would demand similar products to compensate for the original missing products, e.g., cheese and meat of other species, as indicated by the (+) signs]. Substitution imports may have negative macroeconomic outcomes—for example, through a worsening of the country's import/export balance—whereas product substitution may paradoxically benefit other competing sectors.

As shown in Figures 3, 4, brucellosis is a food borne disease that spreads across the food supply chain. It emanates from the production system (which is a part of the supply chain) and ultimately affects humans by way of direct contact, food processing and distribution, and consumption of dairy products. As outlined earlier, Figure 7 starts from the final box of Figures 3, 4 (brucellosis in humans) and further identifies the economic consequences of the disease across society. In simplistic terms, brucellosis in humans may translate into increased morbidity and mortality rate [as shown by (+) signs], which put an increased economic burden [marked by (+) signs] on private and public costs:

– *Public costs:* arrows commencing from these boxes list the type of public costs, mainly incurred by the public health system (i.e., Hospital care for infected people; Informational campaign costs (mass media emissions, printed matter, direct information to communities, etc. to inform about the risk of, and avoid the persistence of, inappropriate practices); and Disease containment (such as food safety control; geographical delimitation of the infected area; field and laboratory analysis; implementation of active strategies to address the disease post-containment etc.)
– *Private costs*: similarly, arrows staring from these boxes list the types of private costs (i.e., Therapies and family assistance (e.g., health care costs of the households, time spent for assistance at home); Loss of revenue due to temporary or permanent disability, which translates into costs for individuals, families and society, depending on the social relevance of the disease; Adoption of good practices in milk/dairy product preparation and consumption; and Psychological suffering due to uncertainty around health status).

The positive and negative economic impacts outlined in Figures 6, 7 typically occur simultaneously (e.g., product substitution may benefit the producers of substitute products). The sum of these costs/benefits should be contrasted with the possibility of avoiding the negative effects (costs) altogether by intervening at an early stage, before disease spread. This is considered to be a benefit of any potential intervention. Economics provide different criteria to categorize intervention costs and related benefits, however a more detailed economic evaluation would focus the costs and benefits the OH-ness and its main dimensions (thinking, planning, working, sharing, learning, systemic organization). Though economic evaluation is not a key aim of this article, the concepts above offer a more precise idea of the complexity of the economic evaluation in the context of One Health, as well as a preliminary agenda for further development of the evaluation process to include OH-ness evaluation as briefly described in this article.

## Discussion

These two case studies, despite their common goal of eradicating or controlling Brucellosis, have quite diverse backgrounds and show different degrees of “One Health” thinking in their respective approaches. In both countries, control was only possible due to constant reminders to farmers and animal owners that the disease could easily spread to humans, together with strict enforcement of legislation. The Malta case study spans a century of failed measures and setbacks, and demonstrates a paradigm shift in the approach to brucellosis eradication over time. The measures implemented ranged from initially relatively isolated actions such as processing of milk through pasteurization (introduced prior to the second World War), to a more sectorial approach adopted in the 1980's. However, it was only upon the adoption of a true “One Health” transdisciplinary approach in the mid-1990's that Brucellosis eradication was successfully achieved and maintained. Enforcement of existing and newly implemented legislation was crucial to this success, and required collaboration by all stakeholders involved including public health, veterinary health, policy-makers and legislators, as well as farmers and consumers' representatives.

The Serbian context differed from the Maltese scenario. Serbia did not have a long history of brucellosis, which was largely sporadic before the mid-1990's and became endemic only as a result of the non-regulated importation of infected cattle and sheep from neighboring countries. There was no cultural resistance to the destruction of potentially infected herds of cattle and sheep, and a greater willingness to accept the scientific rationale for culling within the general population. Additionally, Serbia was able to capitalize on the experience and knowledge of OH thinking adopted in other countries, which explains the somewhat predominantly sectorial approach adopted by the Veterinary Directorate. This proved effective, so that escalation to a fully-fledged transdisciplinary OH approach was not required. It should be noted that the geopolitical conflict in Serbia that ultimately led to “United Nations Security Council resolution 1244” (17) meant that there is a lack of brucellosis-related data for Kosovo after 1999. Given this situation, any challenges regarding brucellosis in Kosovo could not be followed up to the period under study, representing a gap in our assessment.

While brucellosis control was the primary concern in the short term, surveillance and ongoing monitoring remain important medium and long term concerns. This is also reflected in the timing and extent of adoption of OH thinking in the two countries: in Serbia a true OH approach was not required for control, however it is likely to be key to its maintenance in the medium and long term. In Malta, the OH approach was critical in the short term in order to eradicate brucellosis, and together with strict enforcement of legislation remains key to ensuring that the disease does not return. The strength of the OH approach has been tested in recent years. In 2012 a “phantom” herd of unregistered (hence illegal) sheep was identified in Gozo, leading the Veterinary Services Directorate to commence testing and culling of 216 potentially infected sheep. The farmer launched a court case to prevent the remainder of the herd from being depopulated, and a series of appeals and counter-appeals followed with the farmer, the Attorney General, the Police Commissioner and the Director General of Veterinary Services as the main protagonists (33).

There are several lessons to be learnt. In these two case studies, we hope to showcase that One Health initiatives should be applied at the right place, at the right time, with the right people and using the appropriate conditions/infrastructure. One should not adopt a OH approach purely for its own sake or rather wait for all the disciplines to be involved before concrete action is taken. In other words, the One Health transdisciplinary action should not replace but should reinforce the unidisciplinary initiatives taken at the stages of problem identification and action. For example, on the one hand, in the case of Malta, because of the failed attempts at eradication due to fragmented unidisciplinary efforts, only when the Maltese rigorously adopted the OH approach in a transdisciplinary manner, namely by also actively involving the non-scientific community, did they achieve success. The Serbian case study on the other hand showed that the health and agricultural authorities could rely on the aggressive action taken by the Directorate of Veterinary Medicine before moving onto the One Health approach mainly for surveillance prevention. It was the case because most of the infection appeared in animals and number of infected humans was not as high as in Malta. The disease mostly developed in cattle and sheep leaving most of the consequences in economic losses in animal breeding.

The timing of the OH approach is particularly important: in Malta, the right conditions took decades to develop and lessons were painfully learnt over a long period of time, whereas in Serbia the OH approach followed the drastic intervention and leadership of the veterinary department. Sustaining the processes that prevent the re-emergence of brucellosis, however, are likely to require a OH approach.

### Limitations

There are a number of limitations to the method used in this study, including potential bias in the selection of interviewees. In both countries, the fact that the interviewers were “insiders”—members of the outbreak teams—might have influenced the purposive sampling approach used in this study (34). Further limitations include recall bias during interviews, even though we made every attempt to counteract this by triangulating interview data with data mainly from legal documents, archival material from public health and veterinary sources. The application of the NEOH evaluation framework (12) is a novel approach to evaluating One Health and is only recently published. Our experience of using this evaluation tool, is that it requires substantial specific data that is not all available, in particular in view of the retrospective nature of this study. Therefore, some degree of inaccuracy may have resulted in the scoring. The NEOH evaluation tool is based on the systems theory and applies mixed methods, namely descriptive and qualitative with a quantitative scoring. Therefore, capturing the diversities that exist between Malta and Serbia regarding the various sections of the NEOH tool proved to be challenging despite our effort in ensuring rigor throughout the comparative exercise. This case study is one in a series of case studies published under the same research topic that have utilized the NEOH evaluation tool, all providing the first results on One Health Index and One Health Ratio for various One Health initiatives. This will enable validation of the NEOH framework and tool by providing comparisons on the use of the tool and the challenges faced during evaluation and scoring. One Health evaluation should therefore be complemented with other evaluation models for example cost-benefit analysis (costs and benefits expressed in monetary terms) and cost effectiveness analysis (costs vs. project results in units).

## Conclusion

This comparative case study shows that context and timing are key to determining how, when and why a One Health approach should be applied. We conclude that one need not wait for the start of a fully-fledged One Health approach to address a potential health crisis. Instead, each relevant discipline should be on the alert and perform its key responsibilities at an early stage, before scaling up to a transdisciplinary level becomes necessary. Nevertheless, as evident in this article, adopting a OH approach has provided added value not only during the periods of crisis but also in the medium and long term, particularly in the areas of disease prevention and control, surveillance, health promotion and health education. Adopting a OH approach may also translate into cost savings. We therefore propose that OH evaluations should include economic assessments, in order to be able to better understand the optimal use of resources in these situations, thereby justifying funding and political support required.

## Author contributions

SB and SS were responsible for data collection and providing the first framework of the paper. SB, SS, DC, and EL were responsible for preparing the first draft. MC and MA were responsible for the system dimensions, boundaries, elements, relationships, and functioning, as well as the theoretical perspective regarding an economic evaluation and providing advice in the early stages of formulating the case study. All authors contributed in the preparation of the manuscript for submission.

### Conflict of interest statement

The authors declare that the research was conducted in the absence of any commercial or financial relationships that could be construed as a potential conflict of interest.
